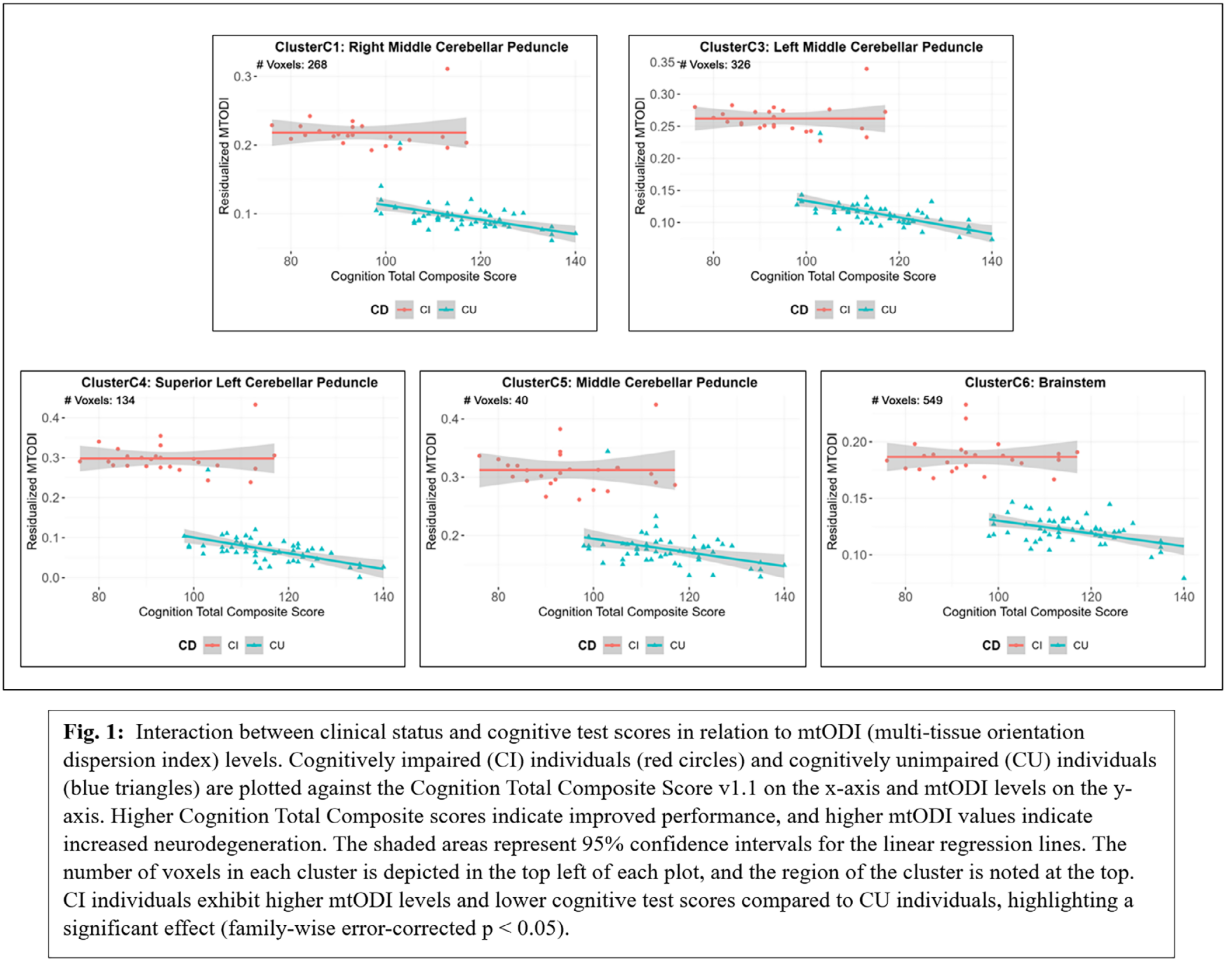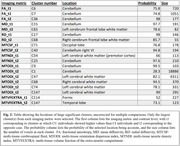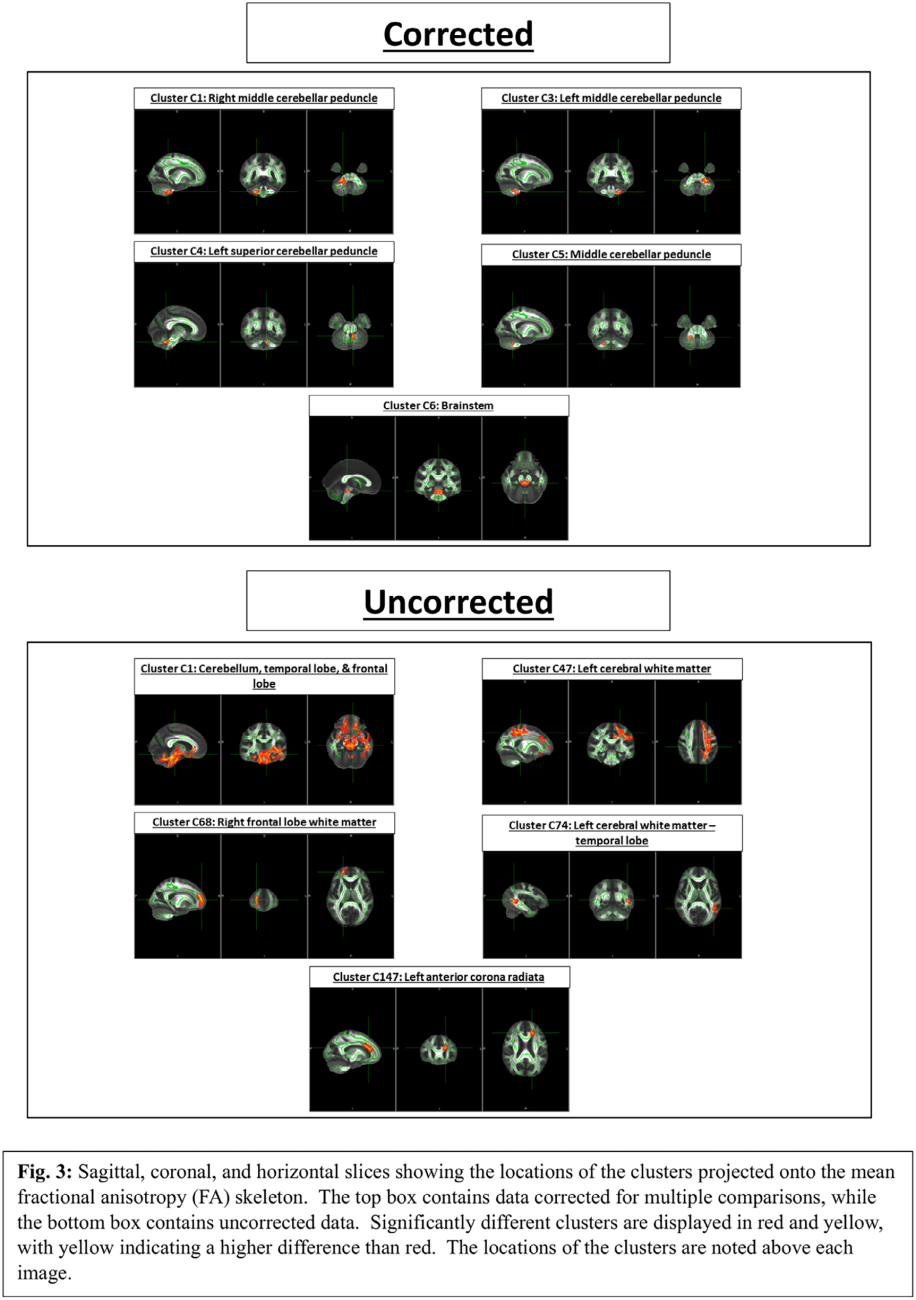# White matter microstructure and cognition in the Alzheimer’s Disease Connectome Project (ADCP)

**DOI:** 10.1002/alz.088285

**Published:** 2025-01-09

**Authors:** John Weston Roberts, Nagesh Adluru, Veena A. Nair, Anusha Adluru, Andrew S Nencka, Shi‐Jiang Li, Richard J Davidson, Ozioma C Okonkwo, Andrew L Alexander, Barbara B. Bendlin, Vivek Prabhakaran

**Affiliations:** ^1^ University of Wisconsin ‐ Madison, Madison, WI USA; ^2^ University of Wisconsin‐Madison, Madison, WI USA; ^3^ Wisconsin Alzheimer's Disease Research Center, School of Medicine and Public Health, University of Wisconsin‐Madison, Madison, WI USA; ^4^ Waisman Laboratory for Brain Imaging and Behavior, University of Wisconsin‐Madison, Madison, WI USA; ^5^ Alzheimer's Disease Research Center, University of Wisconsin‐Madison School of Medicine and Public Health, Madison, WI USA; ^6^ Department of Radiology, University of Wisconsin School of Medicine and Public Health, Madison, WI USA; ^7^ Medical College of Wisconsin, Milwaukee, WI USA; ^8^ University of Wisconsin, Madison, WI USA; ^9^ Center for Health Disparities Research, Department of Medicine, School of Medicine and Public Health (SMPH), University of Wisconsin‐Madison, Madison, WI USA; ^10^ Wisconsin Alzheimer’s Disease Research Center, University of Wisconsin‐Madison, Madison, WI USA; ^11^ Wisconsin Alzheimer's Disease Research Center, University of Wisconsin School of Medicine and Public Health, Madison, WI USA; ^12^ Waisman Center, University of Wisconsin‐Madison, Madison, WI USA

## Abstract

**Background:**

Alzheimer’s disease (AD) has been mainly thought of as a disease involving gray matter changes. However, despite known correlations between white matter integrity and cognition, less is known about how disruptions to white matter during the development of AD underpin cognitive impairment. This study tests the associations between disruptions to white matter along the AD clinical continuum (cognitive unimpaired (CU): cognitive impaired (CI) – Mild Cognitive Impairment (MCI) and AD) and cognition using diffusion tensor imaging (DTI) and multi‐tissue neurite and orientation dispersion and density imaging (mtNODDI) models of the multi‐shell connectome diffusion MRI (ms‐dMRI) data from the Alzheimer’s Disease Connectome Project (ADCP).

**Method:**

Multi‐shell connectome diffusion MRI data from 80 participants (55 CU (32 F, mean age 66.7 +/‐ 6.6; 25 CI (8 F, mean age 73.6 +/‐ 7.3)) in the ADCP were pre‐processed using DESIGNER processing guidelines. Cognition was assessed using the Cognition Total Composite score from the NIH toolbox. Permutation testing was performed using threshold free cluster enhancement and family wise error correction (FWE) and 10,000 permutations. Relationships between cognitive impairment and white matter integrity were deemed statistically significant for FWE corrected p < 0.05. All analyses controlled for age and sex.

**Result:**

Our analysis revealed a significant interaction between clinical status and cognitive test outcomes on the mtODI (multi‐tissue orientation dispersion index) metric, with higher mtODI levels indicating increased neurodegeneration. CU individuals exhibited higher cognitive performance with lower mtODI levels in cerebellar and brainstem regions, whereas CI individuals showed consistently higher mtODI levels in these regions and lower cognitive performance (Figure 1). Moreover, CI individuals presented with lower overall cognitive test scores compared to their CU counterparts. Analysis of clusters uncorrected for multiple comparisons revealed trend‐level differences in frontal and temporal regions (Figures 2‐3).

**Conclusion:**

Results demonstrate that CI individuals who perform worse than CU individuals on cognitive tests also have higher levels of neurodegeneration than CU individuals. Further research is needed to replicate and extend these findings to other tests of cognitive ability, and to help better understand the potential links between cognitive decline in AD with white matter integrity of cerebellar and brainstem regions.